# Acute bilateral mass-occupying lesions in non-penetrating traumatic brain injury: a retrospective study

**DOI:** 10.1186/1471-2482-15-6

**Published:** 2015-01-24

**Authors:** Yu Hu, Hong Sun, Yanqing Yuan, Qiang Li, Siqing Huang, Shu Jiang, Kaili Liu, Chaohua Yang

**Affiliations:** Department of Neurosurgery, West China Hospital, Sichuan University, No 37, Guo Xue Xiang, Chengdu, Sichuan Province China; Department of Orthopedics, Hospital of Chengdu Office People’s Government of Tibetan Autonomous Region, Chengdu, Sichuan Province China; Department of Neurosurgery, Xindu District People’s Hospital of Chengdu, Chengdu, Sichuan Province China

**Keywords:** Bilateral, Head injury, Lesions, Surgery, Traumatic brain injury

## Abstract

**Background:**

Traumatic acute bilateral mass-occupying lesions (TABML) is a common entity in head injury, with high morbidity and mortality. Our aim in this study was to evaluate the benefits of different treatment options and the outcome predictors in patients with TABML.

**Methods:**

From October 2010 to November 2012, a consecutive cohort of patients aged 16–70 years with TABML were retrospectively analyzed based on the clinical and radiological characteristics. Patients with TABML were included if admitted within 24 h after injury and were excluded if they presented with infratentorial lesions, unilateral lesions within the first 24 h after injury, or penetrating head injury. According to their treatment option, patients were divided into three groups: a conservative treatment group, a unilateral surgery group, and a bilateral surgery group. Outcomes were assessed using the Glasgow Outcome Scale (GOS). Binary logistic regression analysis was applied to determine the outcome predictors.

**Results:**

Forty-seven patients (58.8%) had severe injuries (Glasgow Coma Scale score (GCS), 3–8) upon admission, and the overall mortality was 31.3% at 6 months post-injury. The mortality was 55.6% in patients who underwent conservative treatment (N = 18), 17.9% in unilateral surgery patients (N = 39), and 34.8% in the bilateral surgery group (N = 23). In the surgical group, the mortality was 53.3% (8 of 15) in those with a GCS of 3–5, which decreased steeply to 14.9% (7 of 47) of those with GCS ≥ 6. On logistic regression analysis, the absence of pupillary reactivity, disappearances of basal cisterns and conservative treatment were related to higher mortality. A lower initial GCS score was associated with an unfavorable outcome. Midline shift tended to be associated with mortality and an unfavorable outcome, although statistical analysis did not show a significant difference.

**Conclusions:**

TABML is suggestive of severe brain injury. As conservative treatment is always associated with a poorer outcome, surgery is advocated, especially in patients with a GCS score of ≥ 6. Whereas the prognostic value of midline shift might be limited because of the counter-mass effect in TABML, the GCS score, the pupillary reactivity, and particularly, the compression of basal cisterns should be emphasized.

## Background

Traumatic brain injury (TBI) is a heterogeneous entity that encompasses several anatomical patterns, including epidural hematoma (EDH), subdural hematoma (SDH), hemorrhagic contusion (HC), diffuse axonal injury, subarachnoid hemorrhage, intraventricular hemorrhage, and diffuse brain swelling on the macroscopic level [1–4]. EDH, SDH, and HC are common types of acute mass-occupying lesions, which can be observed in various combinations (e.g., SDH with HC), particularly in patients with severe head injury [2, 5–7]. These mass-occupying lesions may also present unilaterally or bilaterally on the computed tomography (CT) scans [8–10].

Traumatic acute bilateral mass-occupying lesions (TABML) is a common entity after contre-coup injury, with an estimated mortality ranging from 20% to 79%; however, the optimal treatment options and the prognostic indicators for TABML are largely unknown [4, 5, 8–12]. Whether all patients with TABML will benefit from surgical intervention and, if surgery is performed, whether surgical intervention should be based on predominantly mass-occupying lesions (unilateral) or bilateral lesions [5, 7–10, 13], remain under discussion. Early prediction of outcomes is not only of great importance in clinical decision-making, but also is useful for stratification of patients for clinical trials and comparison of outcomes of different series [3, 14]. The Glasgow coma scale (GCS) score on admission is a powerful independent predictor in TBI, a fact demonstrated in patients with TABML in previous studies [5, 12]. The midline shift (MLS), another important predictor in TBI, is usually of less significance in bilateral than in unilateral abnormalities because of the counter-mass effect [3–5, 7, 12, 15]. Whether the MLS and other predictors, such as the compression of basal cisterns, correlated with outcomes in TABML has not been extensively studied [3, 5].

In the study reported herein, our aim was to determine the benefits of different treatment options and the outcome predictors in patients with TABML.

## Methods

### Patients

Following approval by the medical ethics committee of the West China Hospital of Sichuan University (Chengdu, China), a consecutive cohort of patients aged 16–70 years with bilateral mass-occupying lesions after TBI was retrospectively enrolled and observed from October 2010 to November 2012. Patients were included if admitted within 24 h after injury. Exclusion criteria were defined as infratentorial lesions or only unilateral lesions within first 24 h after injury that subsequently developed into bilateral lesions [8]. Patients with penetrating head injury or any extracranial injuries that could affect outcomes were also excluded. Patients with limitations to surgical intervention, such as coagulopathy and Do Not Resuscitate (DNR) orders, were also excluded. The demographics, mechanism of injury, pupillary response, admission GCS score, CT characteristics, neurosurgical therapies, and Glasgow Outcome Scale (GOS) score at 6 months after injury were recorded.

### Computed tomography

After initial resuscitation, a CT scan of the head was performed in all patients. MLS (in millimeters) was defined as displacement of the septum pellucidum. Traumatic mass-occupying lesions were noted and their volumes (in milliliters) estimated using the ellipsoid method described previously [3]. The largest lesion responsible for the primary symptoms was regarded as the predominant lesion; accordingly, patients were categorized as EDH with or without HC, SDH with or without HC, and pure HC as previously described [7], with minor modifications. We also scored the following variables on the initial head CT as described elsewhere: the status of basal cisterns (open, partially, and completely obliterated), traumatic subarachnoid hemorrhage (no blood, visible blood) and CT-visible deep lesions (no lesions, visible lesions) [15].

### Treatment

Patients were treated according to guidelines for the management of severe head injury [16] and were divided into three groups based on their treatment option: conservative treatment group, unilateral surgery group, and bilateral surgery group. An intracranial pressure (ICP) probe was implanted in the lateral ventricle or intraparenchymally in 42 cases. Patients who had only ICP monitoring were included in the conservative group. Surgical indications were as follows: (1) signs of brain herniation; (2) clinical deterioration of consciousness and CT-confirmed hemorrhagic progression; (3) obvious mass-occupying effect and the compression of basal cistern; and (4) ICP ≥ 25 mmHg after conservative treatment. The surgical procedures, unilateral or bilateral, were decided on by neurosurgeons according to clinical, radiological, and ICP findings. Operations were performed simultaneously if both lesions needed surgery, or sequentially (operations at separate times) if a significant contralateral lesion or uncontrolled intracranial hypertension (ICP ≥ 25 mmHg) developed after the primary operation developed.

### Outcome

The GOS was assessed at 6 months after injury [1, 3]. A GOS score of ≥ 4 was categorized as a favorable outcome, and a GOS score of ≤ 3 was considered an unfavorable outcome.

### Statistical analysis

The dependent outcome variable was dichotomized as dead versus alive and as unfavorable outcome (GOS 1–3) versus favorable outcome (GOS 4–5) [1]. GCS scores on admission (mean ± standard deviation) were compared using the Mann–Whitney U test in the different treatment groups. The chi-square test was used to compare the survival rate of the different groups. Binary logistic regression analysis was used to further determine the association between outcomes and prognostic indicators, including age, GCS on admission (mild, moderate, severe), pupillary reflexes, MLS, dominant-lesion volume, total lesion volume, cistern compression, traumatic subarachnoid hemorrhage, CT-visible deep lesions, type of predominant lesions, ICP monitoring, and type of treatment (conservative treatment, unilateral surgery, bilateral surgery) [3, 14, 15]. In the regression analysis, age, MLS, dominant-lesion volume, and total lesion volume were treated as continuous or nominal variables, and the remaining independent variables were treated as nominal variables. All data were analyzed with SPSS 16.0 (SPSS Inc., Chicago, IL, USA). Significance was defined by a two-tailed P value of less than 0.05.

## Results

A total of 80 consecutive patients with TABML were included in the retrospective study, the demographic, clinical, and outcome characteristics of whom patients are detailed in Table 1. Upon admission, 19 patients (23.8%) had mild brain injuries (GCS 13–15), 14 (17.6%) had moderate injuries (GCS 9–12), and 47 (58.8%) had severe injuries (GCS 3–8). At 6 months after injury only three patients (3.8%) had been lost to follow-up, leaving 77 patients with a GOS score (2.9 ± 1.6) available. The overall mortality was 31.3% and the rate of unfavorable outcomes was 56.3%.

**Table 1:** **Baseline characteristics of the patients**

Characteristics	No.	Conservative	Unilateral	Bilateral
No. of patients	80	18	39	23
Male gender	66 (82.5)	18 (100.0)	33 (84.6)	15 (65.2)
Age^1^	46.4 (15.7)	43.6 (20.0)	49.2 (14.4)	43.9 (16.7)
Cause of injury				
Traffic	42 (52.5)	10 (55.6)	15 (38.5)	17 (73.9)
Fall	28 (35.0)	6 (33.3)	17 (43.6)	5 (21.7)
Violence	4 (5.0)	0	4 (10.3)	0
Other/missing	6 (7.5)	2 (11.1)	3 (7.7)	1 (4.3)
GCS score on admission^1^	8.5 (3.8)	9.2 (4.3)	9.2 (3.7)	6.7 (3.3)
AIS-head^1^	4.4 (0.8)	4.2 (0.9)	4.3 (0.8)	4.7 (0.6)
Pupillary response				
Present	52 (66.3)	11 (61.1)	31 (79.5)	10 (43.5)
One absent	6 (7.5)	1 (5.6)	2 (5.1)	3 (13.0)
Bilateral absent	21 (26.3)	6 (33.3)	5 (12.8)	10 (43.5)
Outcome				
GOS^1^	2.9 (1.6)	2.6 (2.0)	3.3 (1.5)	2.5 (1.4)
Death	25 (31.3)	10 (55.6)	7 (17.9)	8 (34.8)
Unfavorable (GOS score 1–3)	45 (56.3)	12 (66.7)	16 (41.0)	17 (73.9)
Missing	3 (3.8)	1 (5.6)	2 (5.1)	0

^1^Mean (SD); All other variables: number (%).

GCS, Glasgow Coma Scale; AIS, Abbreviated Injury Scale; GOS, Glasgow Outcome Scale; SD, standard deviation.

The major CT characteristics of TABML are shown in Table 2. Although the dominant-lesion volume (37.6 ± 21.6 ml) and total lesion volume (51.9 ± 28.4 ml) were large, the MLS was often mild (4.0 ± 3.4 mm). SDH with or without HC was the most common type of predominant lesion, accounting for 42.5% of the total of 80 cases, with compression of basal cisterns, subarachnoid hemorrhage, and CT-visible deep lesions being frequently encountered.

**Table 2:** **CT characteristics on admission**

CT characteristics	No. (n = 80)	Conservative (n = 18)	Unilateral (n = 39)	Bilateral (n = 23)
Midline shift				
Presence	67 (83.8)	14 (77.8)	33 (84.6)	20 (87.0)
Mean, mm^1^	4.0 (3.4)	3.6 (3.9)	4.0 (3.0)	4.1 (3.9)
Volume dominant lesion, mL^1^	37.6 (21.6)	27.6 (15.4)	39.4 (18.8)	42.3 (27.6)
Total volume lesions, mL^1^	51.9 (28.4)	41.2 (23.2)	52.4 (27.6)	59.1 (35.5)
Dominant lesion				
EDH	17 (21.3)	1 (5.6)	10 (25.6)	6 (26.1)
SDH	34 (42.5)	7 (38.9)	18 (46.2)	9 (39.1)
HC	29 (36.3)	10 (55.6)	11 (28.2)	8 (34.8)
Compression of basal cisterns				
Open	19 (23.8)	6 (33.3)	12 (30.8)	1 (4.3)
Partially closed	20 (25.0)	2 (11.1)	13 (33.3)	5 (21.8)
Completely closed	41 (51.3)	10 (55.6)	14 (35.9)	17 (73.9)
Subarachnoid hemorrhage	69 (86.3)	14 (77.8)	34 (87.2)	21 (91.3)
CT visible deep lesions	26 (32.5)	7 (38.9)	8 (20.5)	11 (47.8)

^1^Mean (SD); All other variables: number (%).

CT, computed tomography; EDH, epidural hematoma; SDH, subdural hematoma; (HC) Hemorrhagic contusion.

Eighteen patients (22.5%) underwent conservative treatment, 39 (48.8%) unilateral surgery, and 23 (28.8%) bilateral surgery. Bilateral surgery was performed simultaneously in 18 patients (78.3%) and sequentially in five patients (21.7%). The demographic, clinical, and outcome characteristics of the three treatment groups are shown in Tables 1 and 2. In the bilateral surgery group, patients were more likely to present with bilateral absence of pupillary response, large-volume lesions, complete cistern compression, and CT-visible deep lesions. Of the 15 patients with a GCS of 3–5 who underwent surgery, eight (53.3%) died, four (26.7%) survived in a vegetative state, and the remaining three (20.0%) survived with severe disability. However, of the 47 surgical patients with a GCS score ≥ 6, only seven (14.9%) died, three (6.4%) survived in a vegetative state, and eight (17.0%) survived with severe disability.

**Figure 1:**
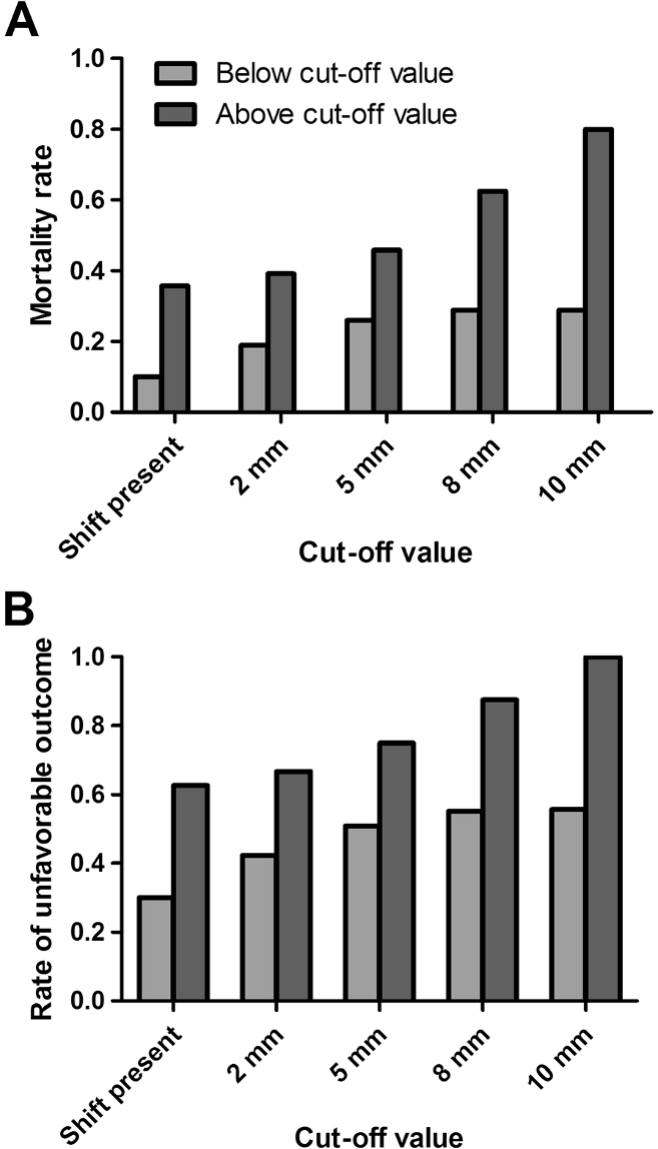
**Outcome and degree of midline shift. (A-B)** mortality rate and percentage of patients with an unfavorable outcome per cut-off value of midline shift.

We found that the absence of pupillary response, the compression of basal cisterns, and the type of treatment were statistically significant predictors of death; the worst GCS score on admission and the compression of basal cisterns both correlated significantly with an unfavorable outcome (Tables 3 and 4). Although the mortality and percentage of patients with an unfavorable outcome were higher in patients with a more severe MLS (Figure 1), as a continuous and nominal variable, MLS did not correlate with the outcomes significantly. Other factors (e.g., the type of dominant lesion) also did not correlate with the observed outcomes.

**Table 3:** **Result of the binary logistic regression analysis for several characteristics and death**

	Death (n = 25)		
Characteristics	OR	95% CI	P
Pupillary response	3.16	1.38, 7.25	0.007
Compression of basal cisterns	3.24	1.04, 10.12	0.043
Type of treatment	0.32	0.13, 0.84	0.020

OR, odds ratio; CI, confidence interval.

**Table 4:** **Result of the binary logistic regression analysis for several characteristics and unfavorable outcome**

	Unfavorable outcome (n = 45)		
Characteristics	OR	95% CI	P
GCS	4.51	1.94, 10.47	0.000
Compression of basal cisterns	2.74	1.17, 6.42	0.020

OR, odds ratio; CI, confidence interval; GCS, Glasgow Coma Scale.

## Discussion

Our study showed that the majority of patients (58.8%) with TABML had severe brain injury. Conservative treatment was more likely associated with higher mortality and treatment failure. Unilateral or bilateral surgery, however, effectively lowered mortality in patients with TABML, especially in those with a GCS score <6. We also found that the GCS score, the pupillary reactivity, and the compression of basal cisterns are important prognostic predictors in TABML. Although MLS is an important prognostic predictor in TBI, our study revealed that it is not an outcome predictor in TABML [3, 15].

From this retrospective study, we cannot simply draw a conclusion that unilateral surgery is better than bilateral surgery based on the mortality alone, because the baseline characteristics of the two groups are not comparable. According to GCS score, the most important predictor of the severity of TBI, we found that bilateral surgery was commonly performed in patients with more severe injuries in comparison with those who underwent unilateral surgery (GCS, 6.7 ± 3.3 VS 9.2 ± 3.7; p < 0.05). Although patients who underwent conservative treatment had higher GCS scores (GCS, 9.2 ± 4.3 VS 6.7 ± 3.3; p < 0.05), a lower survival rate (44.4% VS 65.2%; p < 0.05) was found in comparison with bilateral surgery, indicating that bilateral surgery may be a life-saving procedure for TABML patients [5, 10]. Logistic regression analysis, which adjusted the confounding factors, also confirmed that conservative treatment was related to higher mortality (odds ratio, 0.32; 95% confidence interval, 0.13-0.84).

Our results are consistent with those of several previous studies demonstrating that bilateral surgery is an effective procedure in reducing mortality in patients with TABML [5, 10, 17]. Razack et al. reported a relatively low mortality (20%) in patients with GCS scores of 8.8 ± 0.82 undergoing early simultaneous bilateral craniotomy, attributing the excellent results to the aggressive surgical intervention [5]. Walcott et al. studied a fatal subtype of TABML, bilateral acute SDH, and found that bilateral hemicraniectomy resulted in 33.3% (3 of 9) of patients surviving in-hospital course [10]. This result may be due to surgical decompression being able to reduce intracranial hypertension more efficiently, with shorter duration of intracranial hypertension and fewer interventions [13, 17, 18]. Bilateral decompression has also been reported in patients with bilateral diffuse brain swelling, resulting in an equal mortality and a higher rate of unfavorable outcome in comparison with conservative treatment in a recent randomized trial [18]. The main explanation for the different results between this trial and our study is that TABML represents a more severe entity that is associated with poorer neurological status, higher ICP, and worse outcome [4, 8, 18, 19]. Therefore, conservative treatment is always associated with poor ICP control and treatment failure, as revealed in our series, and aggressive surgical intervention is advocated.

Although bilateral surgery effectively lowered the mortality in TABML, a subset of patients did not benefit from such aggressive intervention [5, 10, 19]. Razack et al. reported five patients with a GCS of 3–5 undergoing bilateral surgery, resulting in 40% mortality and 0% favorable outcome [5]. Walcott et al. found that the in-hospital mortality was 100% in six patients with a GCS of 3–5, and the remaining three patients with a GCS of 6–8 survived [10]. Ucar et al. found that patients with severe TBI with initial GCS scores of 6–8 benefit from decompressive craniectomy, whereas those with GCS scores of ≤5 do not [20]. We agree that patients with an advanced age, a GCS of 3, and bilateral fixed and dilated pupils are strongly identified with a fatal outcome [19]. Our study further confirmed that a GCS score of 3–5 was associated with a bad outcome (53.3% mortality and 0% favorable outcome) even though the aggressive surgical intervention was applied, and we suggest that the aggressive surgery is warranted in patients with a GCS score of ≥ 6 [5, 10, 13, 19, 20].

Our study also demonstrated that the outcome of patients with TABML correlated well with their initial GCS scores and pupillary reactivity. Other studies of TABML have also shown that GCS score was associated with outcome [5, 12]. A study of multiple posttraumatic intracranial lesions found that both a low GCS score and a poor pupillary response predicted a bad outcome [7]. However, because of the early sedation and intubation, the prognostic value of early accessible clinical features (e.g., GCS and pupillary reactivity) is restricted [10, 14]. The status of basal cisterns, an independent predictor unaffected by sedation, is directly related to outcome, as revealed in our study [15, 17]. The fact that the obliteration of basal cisterns was associated with high mortality had previously been demonstrated in patients with bilateral lesions [5, 7, 12]. Taken together, these preoperative prognostic factors, particularly the status of basal cisterns, should be considered as the indications for early aggressive therapy in TABML [5, 21].

More recent studies have revealed that MLS is important in predicting outcome and improving therapeutic decision-making [3, 15]. We studied whether MLS can be useful in predicting outcomes in patients with TABML, and found that it did not correlate with the outcome [5, 7, 9, 10]. The MLS represents pressure difference, which could be absent or mild in TABML because of the counter-mass effect [4, 5, 7, 12]. Then patients may experience intracranial hypertension without obvious MLS being visible on CT. Therefore, when treating patients with TABML, the prognostic value of the MLS might be limited [3, 15, 22].

## Conclusions

TABML is suggestive of severe brain injury with high mortality. Conservative therapy is always associated with treatment failure. However, surgical intervention can lower the mortality effectively and is advocated, especially in patients with a GCS score of at least 6. Although the baseline characteristics of the two surgical groups discussed herein are not comparable, bilateral surgery was always performed in patients with more severe injuries, and is a life-saving option. In treating TABML we should emphasize the importance of the GCS score, the pupillary reactivity, and, particularly, the compression of basal cisterns in outcomes prediction.

## Abbreviations

TABML: Traumatic acute bilateral mass-occupying lesions
GOS: Glasgow Outcome Scale
TBI: Traumatic brain injury
EDH: Epidural hematoma
SDH: Subdural hematoma
HC: Hemorrhagic contusion
CT: Computed tomography
DNR: Do Not Resuscitate
GOS: Glasgow Outcome Scale
ICP: Intracranial pressure.
